# Tonsillar Tuberculosis Simulating Cancer: A Case Report

**DOI:** 10.7759/cureus.70101

**Published:** 2024-09-24

**Authors:** Ilias Benchafai, Sara Aourarh, Abdelfattah Aljalil, Haddou Ammar, Youssef Darouassi

**Affiliations:** 1 Ear, Nose, and Throat, Avicenna Military Hospital, Marrakech, MAR

**Keywords:** cancer, diagnosis, primary, tonsillar tuberculosis, treatment

## Abstract

Tuberculosis is still considered a cause of death, especially in developing countries.

Primary tonsillar tuberculosis in the absence of pulmonary tuberculosis in an immunocompetent patient is a rare entity. Its diagnosis is difficult because it simulates tonsillar cancer, and histopathological examination is often needed for confirmation.

We report the case of a 30-year-old woman with no history of tuberculosis and a normal lung CT. The lack of response to the initial treatment suggested either tumor pathology or germ-specific tonsillitis. The diagnosis of tonsillar tuberculosis was established based on the histological aspects provided by the tonsil biopsy and the QuantiFERON-TB test.

## Introduction

Tuberculosis is still considered a leading cause of death, particularly in developing countries [1]. Extra-pulmonary forms are rare and mainly affect the lymph nodes, bones, urinary system, and abdomen [2]. Tuberculosis of the palatine tonsil represents less than 0.5% of tonsillar pathologies; it is even rarer in its primary form, which is not associated with pulmonary involvement [3].

Its diagnosis is challenging because it mimics tonsillar cancer [3], often requiring histopathological examination for confirmation.

We present a case of a 30-year-old woman with primary tonsillar tuberculosis to provide an update on this rare condition and its differential diagnosis, particularly tonsillar cancer.

## Case presentation

A 30-year-old immunocompetent woman with no particular history who consulted 15 days previously for odynophagia with right otalgia and nightly fever. She was treated with amoxicillin for eight days but without improvement. And given the persistence of her symptoms, her attending physician referred her for an Ear, Nose, and Throat (ENT) consultation.

**Figure 1:**
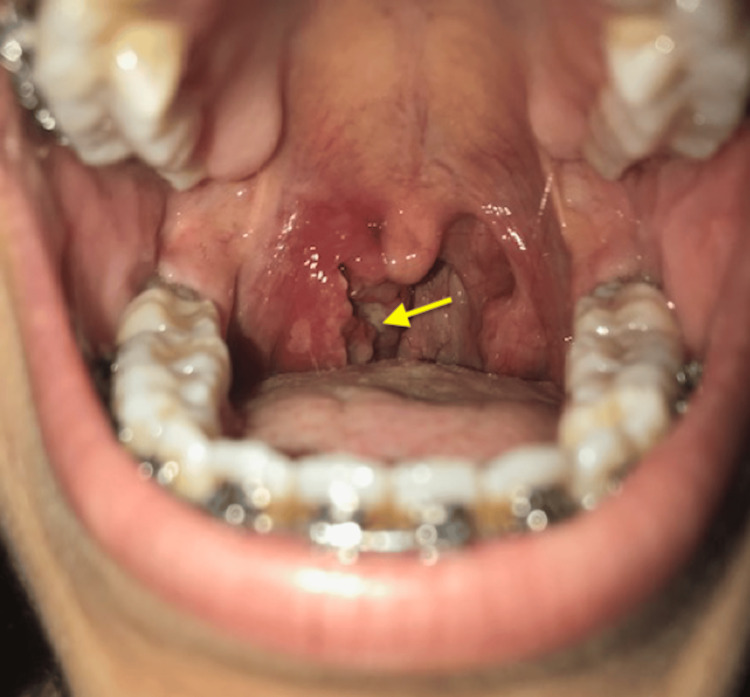
Photograph showing the ulcerative-budding appearance of the right tonsil (yellow-arrow)

Clinical examination revealed a right unilateral hypertrophy of the palatine tonsil with an ulcerous-budding appearance that bleeds on contact (Figure 1).

**Figure 2:**
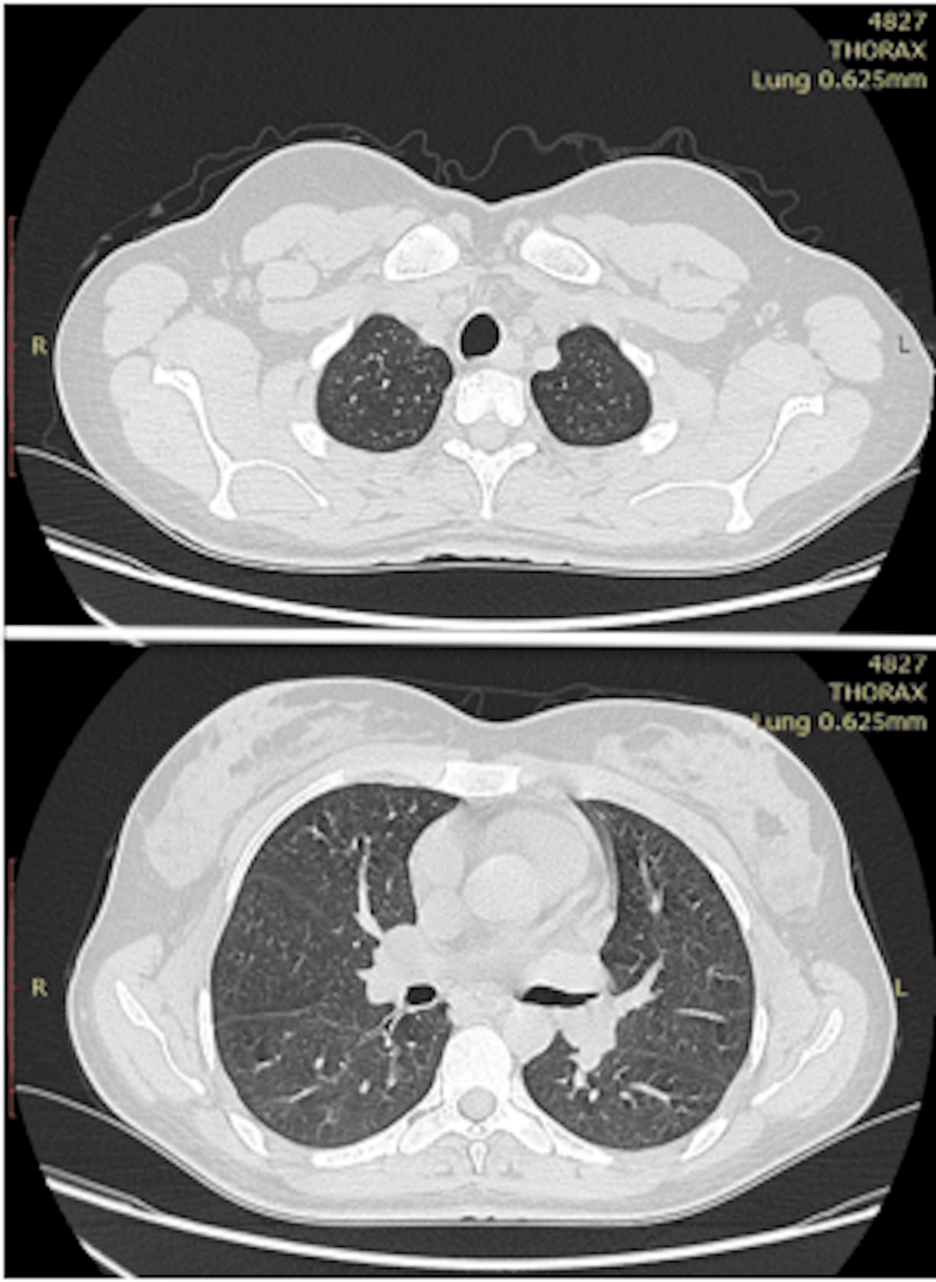
Axial sections of the chest CT show a normal appearance of the lung parenchyma

**Figure 3:**
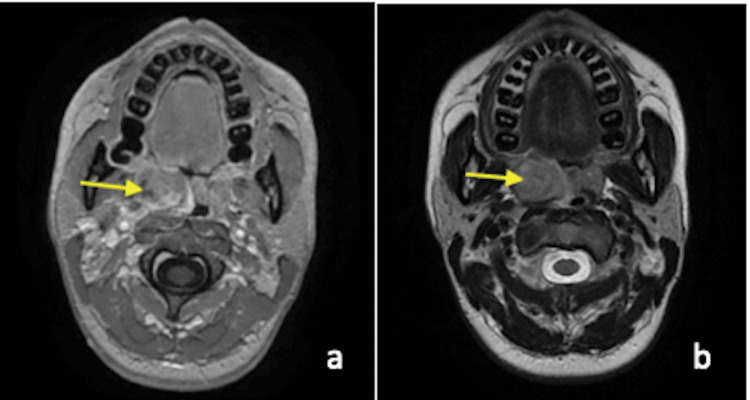
MRI in axial sections, the yellow-arrows show unilateral swelling of the right tonsil with areas of necrosis a: T1 with gadolinium; b: T2

Examination of the neck did not find any palpable lymphadenopathy. The blood tests showed an inflammatory syndrome (VS=55, CRP=24, WBC=13000). The radiological assessment: the lung CT was normal (Figure 2), and the MRI confirmed the swollen appearance of the right palatine tonsil which measured 3 x 3 x 1.5 cm without loco-regional extension and bone lysis (Figure 3).

**Figure 4:**
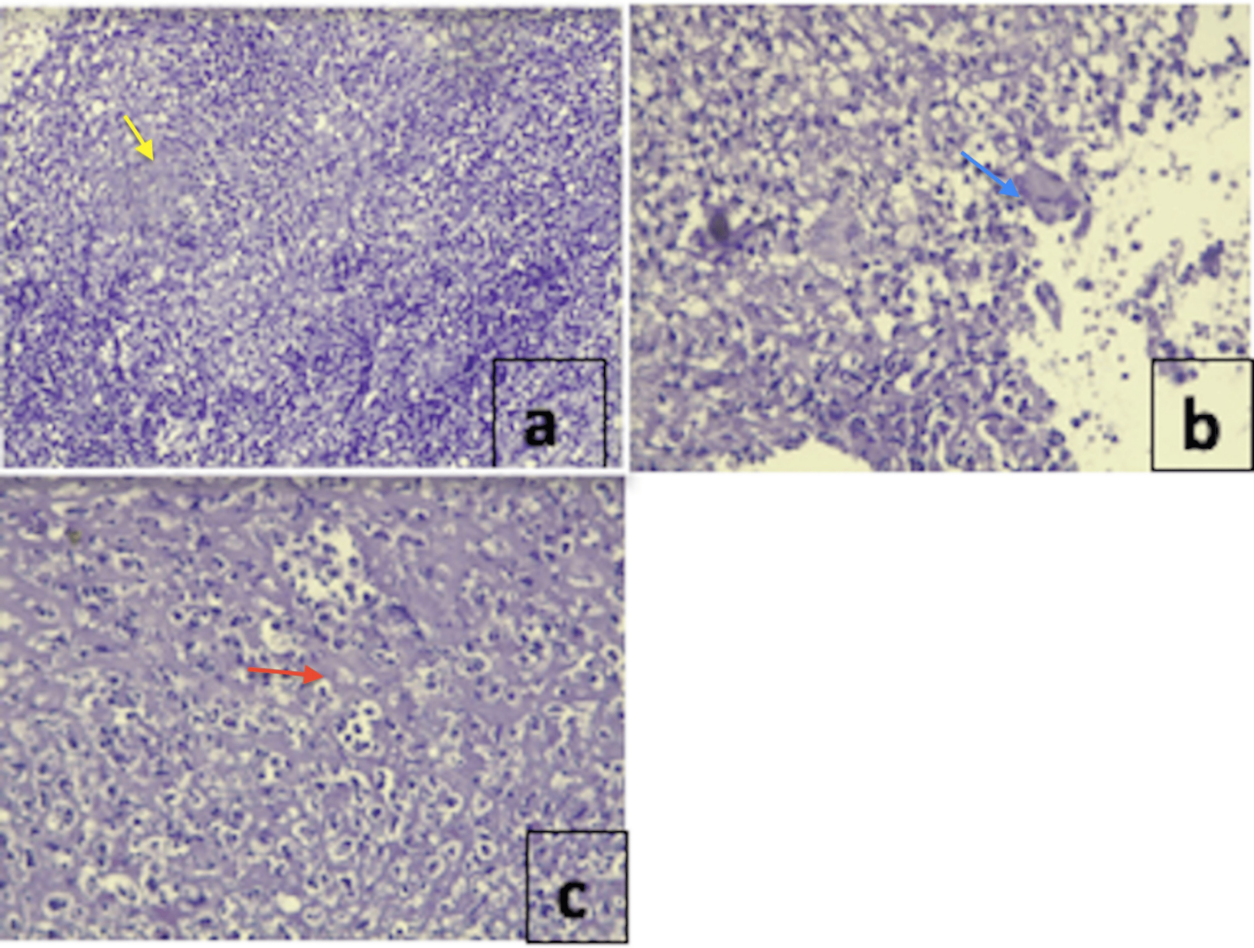
Results a: histological image showing a tuberculoid granulomatous inflammatory reaction Gx10 (yellow arrow = giant cell epithelioid granuloma); b: histological image showing the presence of giant cells (Langhans cells) characteristic of tuberculoid granulomas Gx20 (blue arrow = giant Langhans-type cells); c: histological image showing suppurative necrosis rich in altered polynuclear neutrophils with an outline of caseous-looking necrosis Gx20 (red arrow = outline of caseous necrosis)

Given the suspicious aspect of malignancy of the left tonsil, a biopsy was performed under local anesthesia. The histological study revealed a gigantocellular granuloma with necrosis (Figure 4).

**Figure 5:**
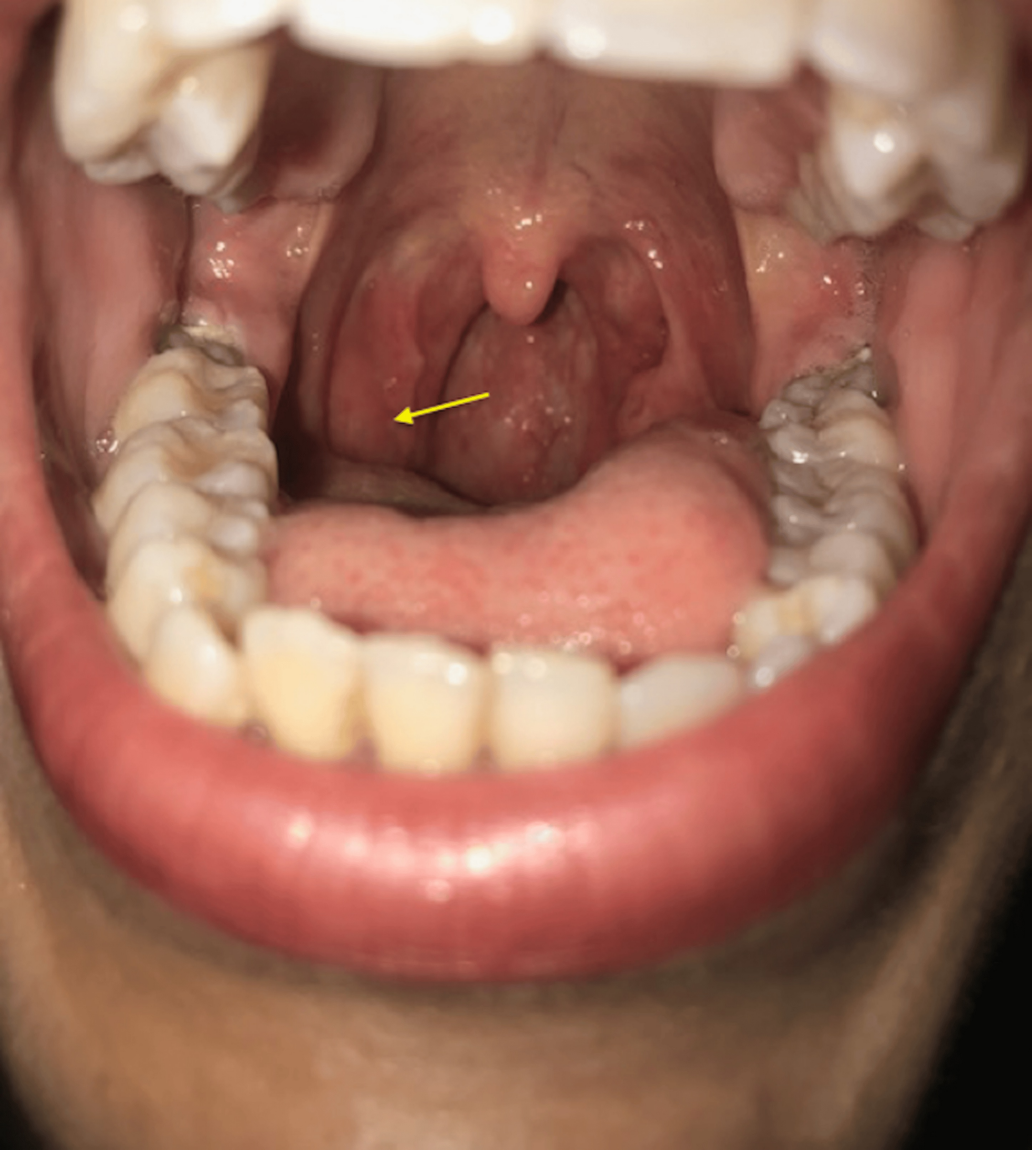
Photograph showing the clinical aspect six months after treatment (yellow-arrow)

The QuantiFERON-TB was positive. The patient then received anti-bacillary treatment according to the following schedule: two months of izioniad + rifampicin + pyrazinamide + ethombutol, then four months of izioniad + rifampicin. One month later, the acid-fast bacilli (AFB) culture came back positive. The control at the end of treatment showed a complete disappearance of symptoms and a normal clinical appearance of the left palatine tonsil (Figure 5).

Finally, the patient did not experience any recurrence of symptoms during the two years of follow-up.

## Discussion

Extra-pulmonary tuberculosis (EPTB) accounts for approximately 20% of tuberculosis (TB) cases [4]. The most common types of EPTB are lymph node tuberculosis, pleural tuberculosis, skeletal tuberculosis, CNS tuberculosis, abdominal tuberculosis, and genitourinary tuberculosis [2]. 

Tuberculosis of the head and neck region makes up nearly 10% of all EPTB cases, with the most common locations being the cervical lymph nodes, followed by the tongue and palate [2]. 

Tonsillar tuberculosis is rare, with only a few cases described in the literature. Tonsillar tuberculosis is classified as primary when it is not associated with pulmonary involvement and secondary when it is associated with documented pulmonary tuberculosis with a positive sputum smear [3].

Primary tonsillar tuberculosis was common in the pre-pasteurization era due to the ingestion of unpasteurized milk infested with Mycobacterium bovis [4]. Our patient was used to regularly consuming unpasteurized milk.

Currently, tonsil tuberculosis is rare, with most cases being secondary cases that result from infection through contact with sputum-containing tubercle bacilli.

Although the oral cavity is the first point of contact for inhaled droplets or aerosols, the incidence of tonsillar tuberculosis is very low. This is thought to be due to saliva's antiseptic and cleansing action, the inherent resistance of the tonsils to tuberculosis infection, the presence of saprophytes in the oral cavity making colonization difficult, and the thick stratified squamous epithelial covering protecting the tonsils [5].

The following conditions can predispose a person to tonsillar tuberculosis: homelessness, incarceration, known contacts, poor dental hygiene, tooth extraction, periodontitis, oral leukoplakia, injection drug use, and HIV infection [6]. However, our patient is immunocompetent, has not had any tooth extractions, has no toxicological habits, does not have known contacts, and does not have poor dental hygiene, periodontitis, or oral leukoplakia.

Symptoms of tonsillar tuberculosis typically include a sore throat, difficulty swallowing, ulceration, and a painless mass lesion, with or without other symptoms of tuberculosis. Swollen cervical lymph nodes may also be present [3,4]. This clinical presentation can resemble that of malignant tumors in the oral cavity, so a histological diagnosis is necessary to rule this out.

The differential diagnosis for tonsillar tuberculosis includes malignant oropharyngeal tumors, which are more common in elderly patients, as well as traumatic ulcers, aphthous ulcers, hematological disorders, actinomycosis, syphilis, and Wegener's disease [7].

A precise ENT clinical examination is necessary to establish a correct diagnosis [8]. 

The biological assessment may appear normal or reveal an inflammatory syndrome. A negative tuberculin skin reaction does not rule out a tuberculous cause. Chest radiography and testing for Koch's bacillus (BK) in sputum or bronchial aspirates, through conventional microbiological examination or by polymerase chain reaction (PCR), can sometimes help diagnose associated lung damage [9]. 

Serological examination for HIV is warranted due to the frequent coexistence of HIV and tuberculosis infections, as well as the significant prevalence of extra-pulmonary forms of tuberculosis in immunocompromised patients [10]. 

A QuantiFERON-TB test, histopathological examination, Ziehl-Neelsen staining, and mycobacterial culture should be performed to establish the diagnosis [2].

The typical features of tonsillar tuberculosis on pathological examination include epithelioid granulomas with caseating necrosis, Langhans, and foreign body giant cells, with or without acid-fast bacilli. The absence of caseating necrosis led to a differential diagnosis with sarcoidosis, but the positive QuantiFERON-TB test supported the diagnosis of tuberculosis [2].

In addition to other specific mandatory laboratory tests for identifying tubercle bacilli, histopathological examination with Ziehl-Neelsen staining should be performed for a definitive diagnosis [2,3]. Even if all confirmatory tests for tuberculosis are negative, epithelioid granulomas with caseating necrosis, Langhans giant cells, and foreign bodies with or without acid-fast bacilli are typical features of tonsillar tuberculosis [7,8]. Therefore, excellent pathology is required for the identification of tonsillar tuberculosis.

The treatment of tonsillar tuberculosis is often medically based on anti-bacillary polychemotherapy; the use of surgery depends on the appearance, clinical impact, and duration of the disease [5].

The anti-tuberculosis treatment includes antibiotics that are both bactericidal and sterilizing (HRSZ) and others that are essentially bacteriostatic (such as ethionamide and ethambutol). The most commonly used protocol consists of rifampicin, isoniazid, pyrazinamide, and ethambutol for two months, followed by rifampin and isoniazid for the next four months, provided there are no symptoms at the end of treatment [5,6]. In disseminated forms, the World Health Organization recommends continuing dual therapy (rifampicin and isoniazid) for seven to 10 months after the initial two-month quadruple therapy.

The objectives of this treatment are to first sterilize the tuberculosis focus using the sterilizing action of the initial quadruple therapy and to prevent secondary resistance by combining several anti-bacillary agents.

The outcome under treatment is often favorable within a few weeks, with the complete disappearance of the lesions, as was the case for our patient [8]. The risk of relapse or persistence of the disease despite well-conducted treatment is 1% [2]. These failures are due to the appearance of BK strains resistant to anti-bacillary treatment [2].

## Conclusions

The case we presented posed a clinical diagnostic challenge. The patient's medical history did not suggest a tuberculosis infection, and the chest X-ray was normal. The lack of response to the initial treatment suggested either tumor pathology or germ-specific tonsillitis.

The diagnosis of tonsillar tuberculosis was established based on the histological aspects provided by the tonsil biopsy and the QuantiFERON-TB test. Isolated primary tonsillar tuberculosis in the absence of pulmonary tuberculosis in an immunocompetent patient is a rare entity, which led us to report this case.
